# PCNA is involved in the EndoQ-mediated DNA repair process in *Thermococcales*

**DOI:** 10.1038/srep25532

**Published:** 2016-05-06

**Authors:** Miyako Shiraishi, Sonoko Ishino, Kotaro Yoshida, Takeshi Yamagami, Isaac Cann, Yoshizumi Ishino

**Affiliations:** 1 Graduate School of Bioresource and Bioenvironmental Sciences, Kyushu University, Fukuoka, Japan; 2 Institute for Universal Biology and University of Illinois at Urbana-Champaign, Urbana, Illinois, USA; 3 Carl R. Woese Institute for Genomic Biology, University of Illinois at Urbana-Champaign, Urbana, Illinois, USA; 4 Department of Animal Science, University of Illinois at Urbana-Champaign, Urbana, Illinois, USA; 5 Department of Microbiology, University of Illinois at Urbana-Champaign, Urbana, Illinois, USA

## Abstract

To maintain genome integrity for transfer to their offspring, and to maintain order in cellular processes, all living organisms have DNA repair systems. Besides the well-conserved DNA repair machineries, organisms thriving in extreme environments are expected to have developed efficient repair systems. We recently discovered a novel endonuclease, which cleaves the 5′ side of deoxyinosine, from the hyperthermophilic archaeon, *Pyrococcus furiosus*. The novel endonuclease, designated as Endonulcease Q (EndoQ), recognizes uracil, abasic site and xanthine, as well as hypoxanthine, and cuts the phosphodiester bond at their 5′ sides. To understand the functional process involving EndoQ, we searched for interacting partners of EndoQ and identified Proliferating Cell Nuclear Angigen (PCNA). The EndoQ activity was clearly enhanced by addition of PCNA *in vitro*. The physical interaction between the two proteins through a PIP-motif of EndoQ and the toroidal structure of PCNA are critical for the stimulation of the endonuclease activity. These findings provide us a clue to elucidate a unique DNA repair system in Archaea.

DNA is always under threat of change or loss of genetic information by endogenous or exogenous influences. To maintain genome integrity for their offspring, and to prevent disorder of a cell system, all living organisms have evolved DNA repair mechanisms. One of the predominant DNA damages encountered by cells is base deamination^1^. Deamination of cytosine, adenine, and guanine gives rise to uracil, hypoxanthine, and xanthine, respectively. Uracil and hypoxanthine are also mis-incorporated into the nascent DNA strand by DNA polymerase during replication. If these bases remain in DNA, they lead to point mutations during replication due to wrong base pairing. Generally, the deaminated bases are released from the DNA strand by a lesion-specific DNA glycosylase. The resultant apurinic/apyrimidinic (AP) site is recognized and the DNA backbone is cut by AP endonuclease (APE). DNA polymerase synthesizes the new strand and DNA ligase fills the nick. This repair pathway is called base excision repair (BER)^2^,^3^. Uracil-DNA glycosylase (UDG), which removes uracil, is the most conserved DNA glycosylase in all domains of life, Bacteria, Archaea and Eukarya. The UDGs are now classified into five families, based on their substrate specificity and amino acid sequence motifs in the active site, although the UDGs form a single protein superfamily with a common structural fold^4^,^5^, suggesting that the repair of damaged bases have been divergently evolved.

Besides the fundamental DNA repair systems like BER, organisms thriving in extreme environments are thought to have developed efficient DNA repair systems, since harsh conditions such as high temperature, ionizing radiation, and acidic/basic pH promote DNA damage. Endonuclease Q (EndoQ) is an enzyme recently isolated from the hyperthermophilic archaeon, *Pyrococcus furiosus*^6^. This enzyme (PfuEndoQ) recognizes uracil, hypoxanthine, AP site and xanthine, and cleaves the phosphodiester bond at the 5′ side of the damaged base, leaving 5′ phosphate and 3′ hydroxyl groups. EndoQ is conserved in the *Thermococcales* (the genus *Pyrococcus* and *Thermococcus*) and some methanogenic archaea, but it does not belong to any of the previously described groups of DNA repair proteins. The homolog from *Thermococcus kodakarensis* (TkoEndoQ) also exhibited the same biochemical properties^6^. Furthermore, it is of note that a homolog is found in a few bacteria, but so far not in any eukaryotic organism.

Biochemical characterization of EndoQ showed that it is involved in the damaged DNA base repair system. However, there is no evidence for how EndoQ functions in this process. Although another hypoxanthine specific endonuclease, Endonuclease V (EndoV), is considered to function in removing deaminated adenine in *P. furiosus*, as well as in *E. coli* and other prokaryotes^7^, our *in vitro* analyses predicted that EndoQ and EndoV are not involved in the same repair pathway, but rather work independently^8^. Furthermore, PfuEndoQ is expected to act more effectively on hypoxanthine-containing DNA than EndoV from *P. furiosus* cells^8^.

To address the question of how EndoQ works in the repair of damaged bases in DNA in the *Thermococcales*, we have been searching for its interaction partners. Proliferating cell nuclear antigen (PCNA) plays an essential role in DNA transactions, including replication, repair, recombination, and cell cycle control^9^. PCNA is a ring-shaped trimeric complex. The central hole of the PCNA ring encircles double-stranded DNA to provide a scaffold to many proteins that acts on DNA, and it is called the clamp molecule. The β-clamp (identified as the β subunit of DNA polymerase III) in Bacteria has same functions as PCNA^10^. Proteins interacting with PCNA possess a consensus sequence motif called PIP (PCNA-interacting protein) box (Qxx*h*xx*aa*: x, any amino acid; *h*, hydrophobic residues; *a*, aromatic residues)^11^,^12^. A similar motif to PIP box is also conserved as a β-clamp binding sequence in Bacteria^10^. In this study, we found a PIP box-like motif at the C-terminal region of EndoQ. With respect to proteins that are involved in the early steps of the BER pathway from Archaea, previous studies showed that PCNA interacts with UDG and APE and enhances the glycosylase activity of UDG and the 3′-5′ exonuclease activity of APE in *P. furiosus*^13^,^14^. It has also been shown that UDG from *Sulfolobus solfataricus*^15^ and from *Pyrobaculum aerophilum*^16^ interact with their PCNA. Hence, the PIP box-like motif in the EndoQ protein implies the possibility that PCNA is involved in EndoQ function. Here we report the physical and functional association of PCNA with EndoQ *in vitro* and propose a repair pathway in the *Thermococcales*.

## Results

### EndoQ homologs have a PIP-box motif at the C-terminus

**Figure 1:**
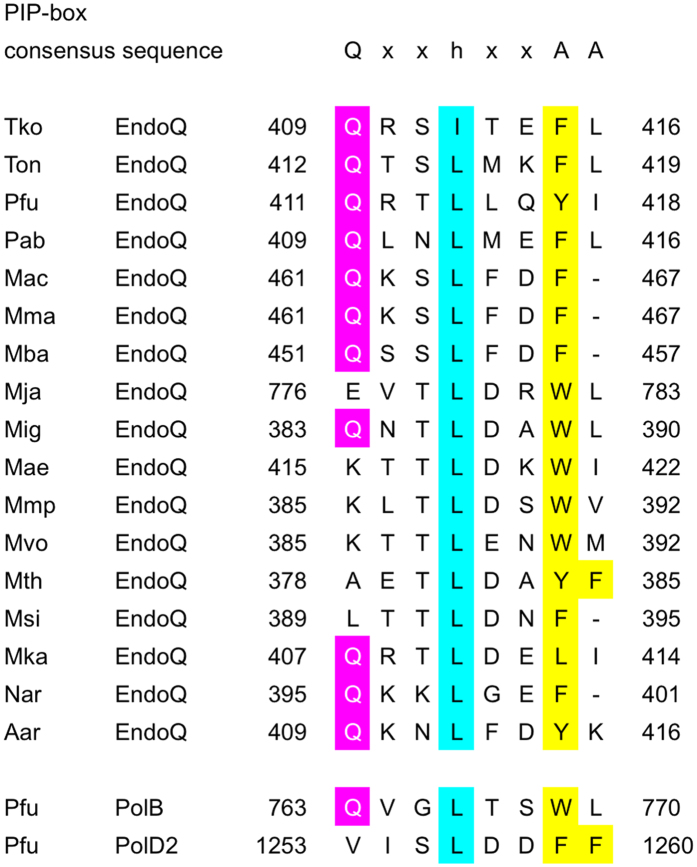
Putative PIP boxes in EndoQ homologs in Archaea. PIP box consensus sequence is shown on the top of the panel: Q, a glutamine residue (magenta background); x, any amino acid; h, hydrophobic residues (L, I or M; cyan background); A, aromatic residues (F, Y or W; yellow background). The residues consistent with the PIP box consensus sequence are in the same color. Tko, *Thermococcus kodakarensis* KOD1 (BAD85076); Ton, *Thermococcus onnurineus* NA1 (ACJ15906); Pfu, *Pyrococcus furiosus* DSM 3638 (AAL81675); Pab, *Pyrococcus abyssi* GE5 (CAB49547); Mac, *Methanosarcina acetivorans* C2A (AAM04083); Mma, *Methanosarcina mazei* Go1 (AAM31501); Mba, *Methanosarcina barkeri* str. Fusaro (AAZ70511); Mja, *Methanocaldococcus jannaschii* DSM 2661 (AAB98023); Mig, *Methanotorris igneus* Kol 5 (AEF96206); Mae, *Methanococcus aeolicus* Nankai-3 (ABR56895); Mmp, *Methanococcus maripaludis* C5 (ABO35878); Mvo, *Methanococcus voltae* A3 (ADI37103); Mth, *Methanothermobacter thermautotrophicus* str. Delta H (AAB85783); Msi, *Methanobrevibacter smithii* ATCC 35061 (ABQ87334); Mka, *Methanopyrus kandleri* AV19 (AAM01639); Nar, *Nanoarchaeota archaeon* SCGC AAA011-K22 (WP_039268096); Aar, *Aenigmarchaeota archaeon* JGI 0000106-F11 (WP_042665925). The sequences were aligned by ClustalW2 (http://www.ebi.ac.uk/Tools/msa/clustalw2/). PIP boxes of DNA polymerase B and DP2 from *P. furiosus* are shown on the bottom. Positions of the motifs are indicated by the amino acid number on the left (start) and right (end) of the sequences.

An alignment of the amino acid sequence showed that most EndoQ homologs from Archaea, except for the *Methanomicrobiales,* have PIP box-like motifs at their C-terminal region (Fig. 1). Thus we assumed that EndoQ proteins would interact with PCNA through the motifs. It is also of note that the *endoQ* gene is present in Bacteria, such as *Bacillus subtilis* and *Disulfovivrio* sp., although EndoQ is mainly conserved in Archaea^6^. It is yet to be determined if these *endoQ* genes are expressed in the bacterial cells and have a function to cleave the DNA at the damaged site. However, the consensus sequences of the β-clamp binding motif^10^ were found in the C-terminal region of the putative sequences of the bacterial EndoQ homologs. It will, therefore, be interesting to investigate if the physical and functional interactions between EndoQ and the clamp molecules from Bacteria, even though PCNA and β-clamp are thought to have evolved independently (see Supplementary Fig. S1).

### Preparation of TkoEndoQ and TkoPCNA1 proteins

**Figure 2:**
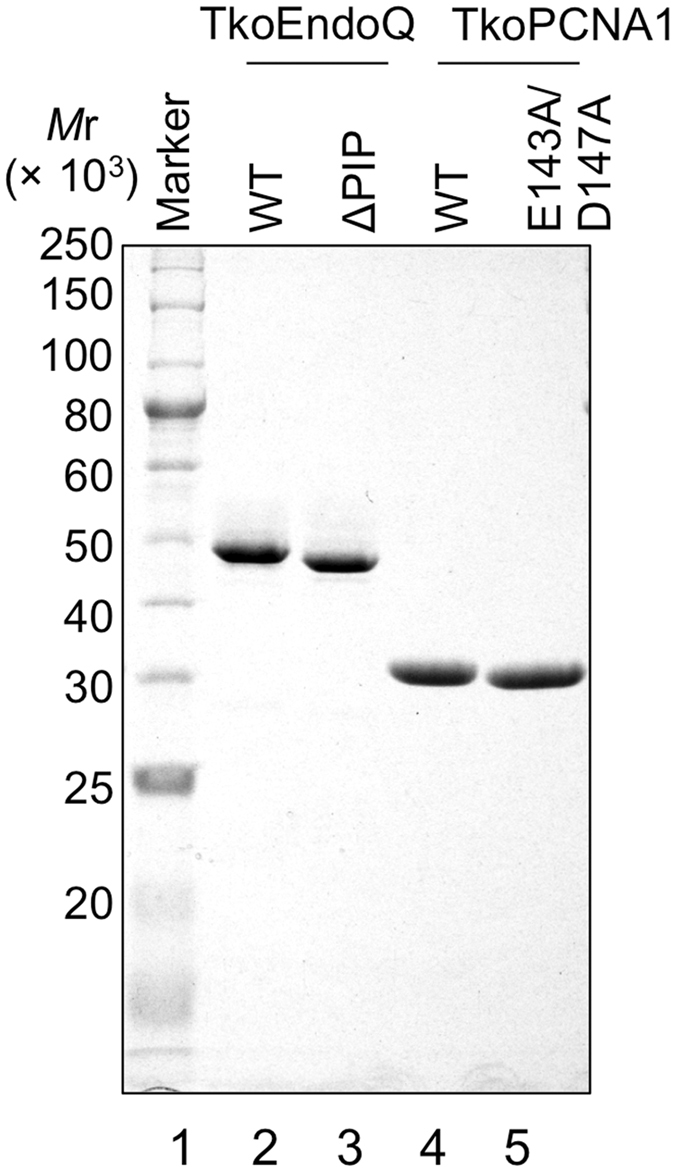
Preparation of the recombinant proteins. The protein marker (NEB, P7703, lane 1) and 1 μg of each purified protein (TkoEndoQ^WT^, lane 2; TkoEndoQ^ΔPIP^, lane 3; TkoPCNA1^WT^, lane 4; and TkoPCNA1^E143A/D147A^, lane 5) were subjected to SDS-12% PAGE followed by Coomassie brilliant blue (CBB) staining. The sizes of the marker are shown on the left of the panel.

To investigate the interaction between EndoQ and PCNA from *T. kodakarensis*, we prepared the mutant EndoQ with truncation of the PIP-box-like sequence and mutant PCNA with point mutations at the interface of the protomers for disruption of the ring structure. We deleted the amino acids from position 409 to 421 for TkoEndoQ, and designated it TkoEndoQ^ΔPIP^. It is known that the D143A/D147A mutant of PfuPCNA cannot form a stable ring structure in solution^17^, and therefore, the corresponding E143A/D147A mutations were made in TkoPCNA1. *T. kodakarensis* has two PCNAs, and PCNA1, but not PCNA2, is essential for cell viability^18^,^19^. Recombinant proteins expressed in *E. coli*, i.e., TkoEndoQ^WT^ (MW: 48080.3), TkoEndoQ^ΔPIP^ (MW: 46491.5), TkoPCNA1^WT^ (MW: 28239.4) and TkoPCNA1^E143A/D147A^ (MW: 28137.4) were purified to near homogeneity (Fig. 2). To confirm the disruption of the ring structure of TkoPCNA1^E143A/D147A^ in solution, purified TkoPCNAs were subjected to gel filtration analysis (see Supplementary Fig. S2). Each protein eluted as a single peak, but the elution positions were different. The molecular weight estimation of TkoPCNA1^E143A/D147A^ was 37.3 k, while TkoPCNA1^WT^ was 99.1 k from the elution profiles. It is already known that PCNA molecules are eluted slightly earlier than the calculated molecular weights^17^. This result suggests that TkoPCNA1^E143A/D147A^ exists as a monomer in solution even at a high concentration (160 μM). Maintenance of the structural conformation of TkoEndoQ after deletion of the C-terminal PIP region was supported by the comparison of the CD spectra from TkoEndoQ^WT^ and TkoEndoQ^ΔPIP^. Two spectra that were almost superimposed were obtained from the two proteins (see Supplementary Fig. S3). Further experiments were performed using these purified proteins.

### Physical interaction between TkoEndoQ and TkoPCNA1

**Figure 3:**
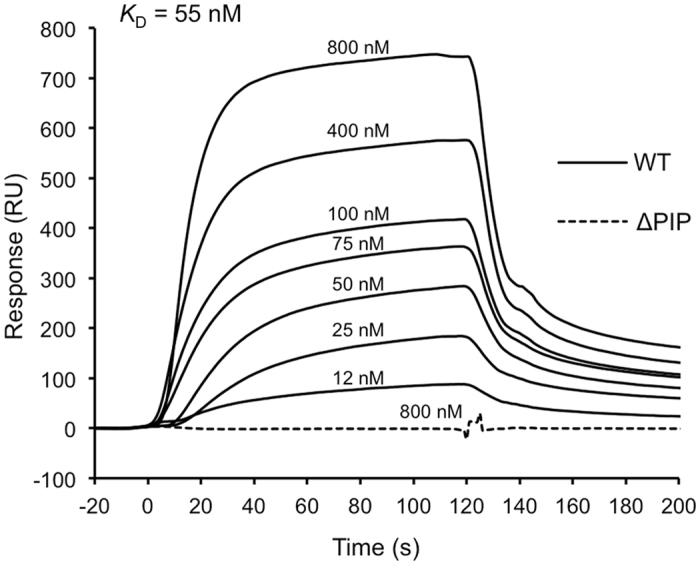
Physical interaction between TkoEndoQ proteins and TkoPCNA1. Surface plasmon resonance (SPR) analysis was conducted using a BiacoreJ system. TkoEndoQ^WT^ at various concentrations (12, 25, 50, 75, 100, 400 and 800; solid lines) and TkoEndoQ^ΔPIP^ (800 nM, a dotted line) were injected on the chip immobilized with TkoPCNA1^WT^ for 120 s in 10 mM HEPES, pH7.4, 0.005% Tween20 and 0.4 M NaCl. The sensorgrams from TkoEndoQ^WT^ were fitted to the 1:1 reaction model to calculate *K*_D_.

To investigate whether TkoEndoQ physically binds TkoPCNA1, surface plasmon resonance (SPR) analysis was performed using the purified proteins. As shown in Fig. 3, TkoEndoQ showed the positive sensorgram against the immobilized TkoPCNA1, and the responses increased in a protein concentration-dependent manner. The *K*_D_ value for the interaction between the two proteins was 55 nM, which was calculated from the sensorgrams of seven different concentrations of TkoEndoQ. On the other hand, TkoEndoQ^ΔPIP^ did not show any response with TkoPCNA1 even at a high concentration up to 800 nM. These results clearly indicated that the PIP-box located in the C-terminus of TkoEndoQ is essential for its interactions with TkoPCNA1. In this experiment, TkoPCNA1 was fixed on a sensorchip at less than 2 μM, in which TkoPCNA1^WT^ exists as a monomer in solution as we showed previously^18^. Therefore, TkoEndoQ should binds to the monomeric form of TkoPCNA1 as observed in many other PCNA binding proteins.

### Stimulation of endonuclease activity of TkoEndoQ by TkoPCNA1

**Figure 4:**
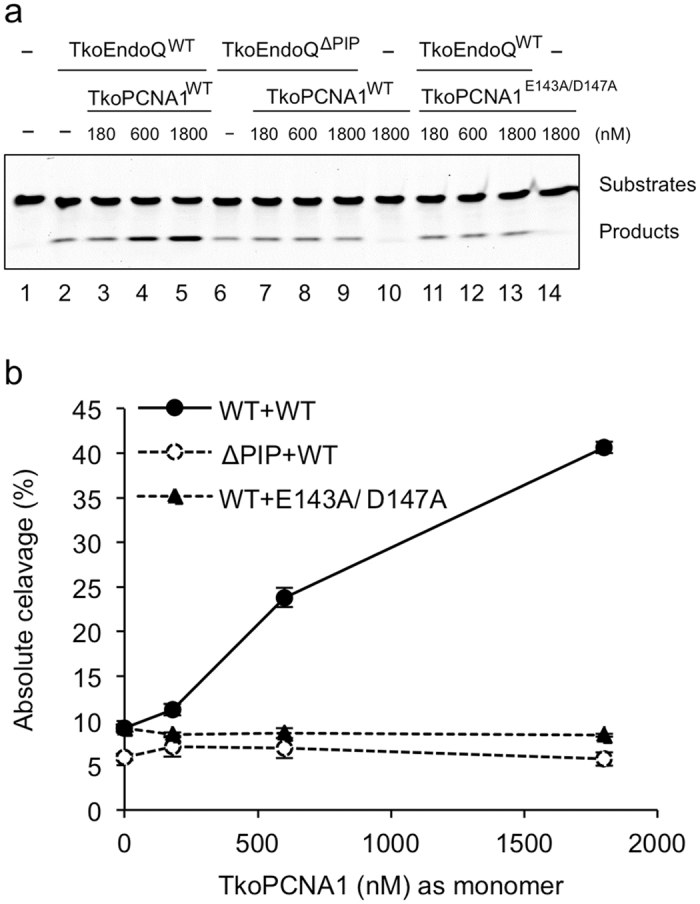
TkoPCNA1 enhances TkoEndoQ cleavage activity on dI-containing DNA. 5′-Cy5-labeled 45-I25 dsDNA (5 nM) were incubated at 75 °C for 10 min, as described in the Materials and Methods section. (**a**) The incubations of 10 nM TkoEndoQ^WT^ (lanes 2–5 and 11–13) or TkoEndoQ^ΔPIP^ (lanes 6–9) were conducted without TkoPCNA1 (lanes 1, 2 and 6) or with TkoPCNA1^WT^ (lanes 3–5 and 7–10); TkoPCNA1^E143A/D147A^ (lanes 11–14) at different concentrations as monomer (0 (−), 180, 600 and 1800 nM, as shown on the top of the panel). TkoEndoQ were not added in lanes 1, 10 and 14. Cleavage products were separated by 8-M urea-12% PAGE. (**b**) Resulting band intensities from Fig. 4a experiments were quantified with ImageQuant TL (GE healthcare). “Absolute cleavage (%)” indicates the percentages of cleaved products in the total DNA bands per lane. Values are averages evaluated from three independent experiments. Combination of the proteins for reactions: TkoEndoQ^WT^ and TkoPCNA1^WT^, a solid line with closed circulars; TkoEndoQ^ΔPIP^ and TkoPCNA1^WT^ a dotted line with open circulars; and TkoEndoQ^WT^ and TkoPCNA1^E143A/D147A^, a dotted line with closed triangles.

To gain an infromation of how the physical interaction between EndoQ and PCNA contribute to DNA repair and the genome integrity, a cleavage assay using TkoEndoQ and TkoPCNA1 was conducted. Using an assay condition, in which TkoEndoQ^WT^ exhibited 9% cleavage on one deoxyinosine (dI)-containing DNA, the rate of the cleavage was increased in a TkoPCNA1 concentration-dependent manner (Fig. 4a, lanes 2 to 5). When TkoPCNA1^WT^ was added at 180, 600 and 1800 nM (60, 200 and 600 nM; as a trimer) to the reaction, the rate of the cleavage was increased to 11%, 24% and 41%, respectively. Conversely, when TkoEndoQ^ΔPIP^ or the monomeric mutant of TkoPCNA1^E143A/D147A^ was used, this stimulation was not detected. Notably, the TkoEndoQ^ΔPIP^ mutant showed 6–7% cleavage either with or without TkoPCNA1^WT^ (Fig. 4a, lanes 6 to 9). The TkoPCNA1^E143A/D147A^ mutations did not affect the cleavage activity of the TkoEndoQ^WT^ (Fig. 4a, lanes 11 to 1413). These results support our observation that the TkoPCNA1 stimulated endonuclease activity of EndoQ depending on the presence of the PIP box-like motif, and the ring structure of the PCNA is important for this function. The SPR experiment shown above supports that TkoEndoQ specifically binds to the monomeric form of TkoPCNA1. However, the ring structure TkoPCNA1 is necessary to stimulate the endonuclease activity of TkoEndoQ as shown here, although one EndoQ molecule on one PCNA ring is enough and is possible to access the reaction site on DNA. Generally, PCNA binding proteins can bind a PCNA protomer, but needs a ring-structured PCNA to encircle DNA for their functional interactions.

### Interaction of EndoQ and PCNA is conserved in the *Thermococcales*

To confirm that the EndoQ-PCNA interaction is conserved in the *Thermococcales*, purified PfuEndoQ and PfuPCNA were used for the interaction/stimulation analyses (see Supplementary Fig. S4). PfuPCNA clearly stimulated the endonuclease activity of PfuEndoQ on the dI-containing DNA by 6–7 fold (see Supplementary Fig. S5). Because the purified TkoEndoQ protein has more non-specific binding property to DNA and proteins as compared with PfuEndoQ, a higher salt concentration (0.4 M NaCl) was required for its manipulation *in vitro*. In addition, the endonuclease activity of TkoEndoQ showed more salt-resistance than PfuEndoQ. From these differences, the cleavage assay for PfuEndoQ was performed under reduced concentration of NaCl (0.18 M). To confirm that the EndoQ and PCNA were in the same complex in the cells, an immunoprecipitation experiment was performed using extracts from exponentially growing *P. furiosus* cells and antibodies raised against TkoEndoQ and PfuPCNA (a cross-reactivity of PfuEndoQ against the anti-TkoEndoQ antibody was confirmed before this IP experiment). PfuEndoQ and PfuPCNA co-precipitated with anti-TkoEndoQ or anti-PfuPCNA antibody, respectively (see Supplementary Fig. S6).

## Discussion

We presented here that EndoQs from *T. kodakarensis* and *P. furiosus* interact with PCNA, and therefore, EndoQ may be involved in the replication-associated repair pathway at the replication fork, as proposed previously for *P. furiosus* UDGs^13^. It was also reported that APE of *P. furiosus* interacts with its cognate PCNA both *in vivo* and *in vitro*^14^. Furthermore, an efficient BER process, in which UDG and APE are bound simultaneously to the same PCNA trimer, and an efficient progress of the repair process including the sequential cleavages of the glycosyl bond of uracil and the diester bond has been proposed^14^,^20^. The multiprotein complex, including UNG2, APE1 (AP endonuclease), XRCC1, Polα, β, δ, ε, DNA ligase 1, and DNA dependent protein kinase, was also isolated from the nuclei of human cycling cells^21^. These reports indicate that archaea, possessing EndoQ may have more efficient repair systems for the damaged bases during replication fork progression.

It is now well known that many of the family B DNA polymerases from the hyperthermophilic archaea, including *P. furiosus* PolB, specifically recognize uracil bases in the template strand and stall complementary strand synthesis. This property of the archaeal DNA polymerases has been implicated as an intrinsic activity for the removal of uracil bases^22^,^23^,^24^. It is also possible that PolB and UDG bind to the same PCNA ring to switch at the uracil site. In addition to UDG and PolB, dUTPase, which probably contributes to precise DNA replication by preventing dUTP incorporation in the cells, is also found in *P. furiosus*^25^. Functional associations of PolB, UDG and dUTPase were proposed as a complex named ‘uracilosome’ for the efficient escape from uracil under hyperthermophilic conditions^26^, although the complex has not been isolated from any hyperthermophilic archaea. In addition to these molecules, we propose here that EndoQ is a member of the “uracilosome” in the *Thermococcales* and likely in other archaea harboring its homologs. Uracil is produced by the frequently occurring deamination of cytosine, especially at high temperatures, and therefore, it is possible that the hyperthermophilic archaea acquired the efficient prevention system to alleviate mutations by cytosine deamination.

We showed here that EndoQs also interact with PCNA likely through the PIP-box-like motif in their C-terminal regions. The predicted PIP-boxes are QRSITEFL in *T. kodakarensis* and QRTLLQYI in *P. furiosus,* respectively, and these are typical consensus sequences of the PIP-box. The location of these sequences at the very C-terminus is also typical among the PCNA-binding proteins. In the case of UDG and APE in *P. furiosus,* PCNA binding sites are not in the terminus, but the internal part of the proteins, and shorter versions of the PIP-box, AKTLF in UDG^13^ and TIAGI^14^ in APE, were proposed, as well as for DNA ligase, which also has a shorter version of the PIP box, QKSFF, in its internal site^27^. The apparent *K*_D_ values, calculated from the SPR analysis were 55 nM for TkoEndoQ and TkoPCNA1. These results suggest that EndoQ has stronger affinity to PCNA as compared with UDG and APE. The apparent *K*_D_ values for PfuUDG and PfuAPE with PfuPCNA are 220 nM and 1 μM, respectively^13^,^14^. In consideration with a very close relationship between *P. furiosus* and *T. kodakarensis,* EndoQ may mainly work for removal of uracil and also other damaged bases in the *Thermococcal* cells.

**Figure 5:**
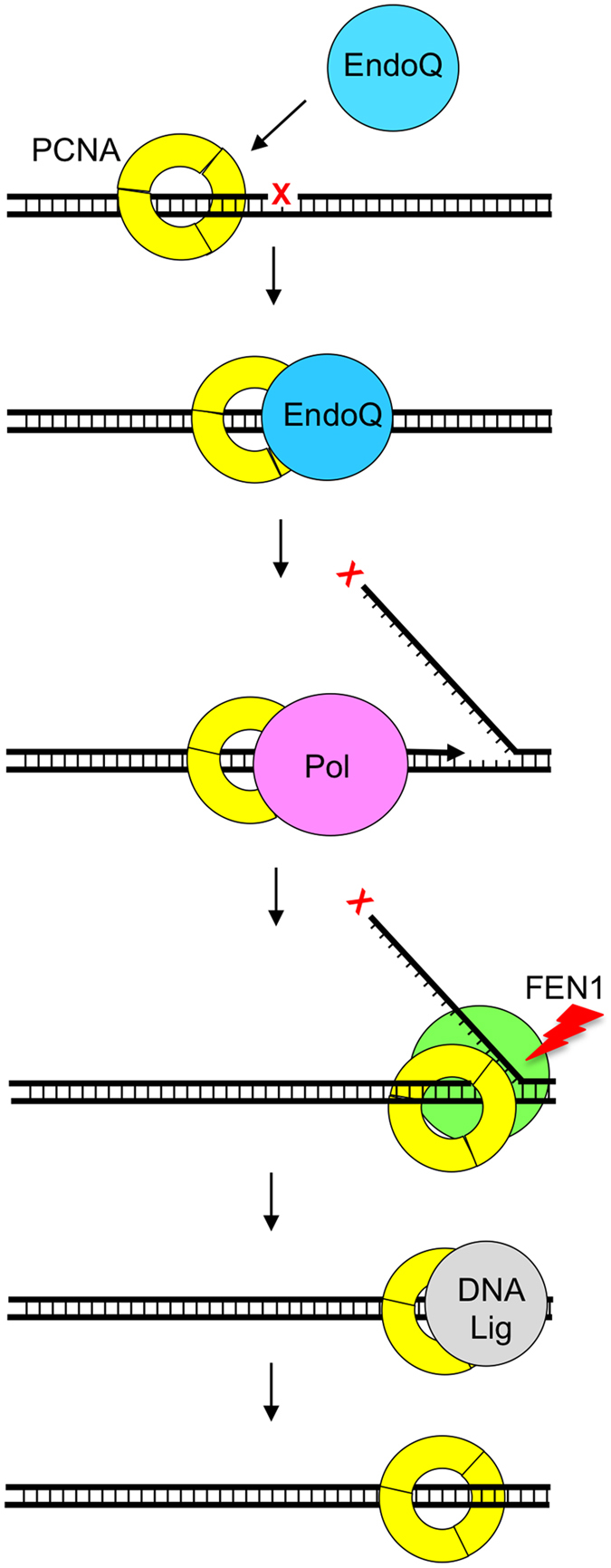
Schematic representation of the model of PCNA-dependent process of EndoQ-mediated repair. PCNA-bound EndoQ recognizes a damaged base in DNA and makes as incision on its 5′-side. PolB on the same PCNA synthesizes a new DNA strand coupled with the 5′–3′ strand displacement of the forward strand. Fen1 cuts off the resulting flap and DNA ligase seals the nick.

The *Thermococcales* have one family B DNA polymerase (PolB) and one family D DNA polymerase (PolD), which are supposed to be replicative DNA polymerases^28^. However, genetic analyses showed that the *polB* gene can be disrupted in *T. kodakarensis* genome and it may mainly work for repair processes^29^. Our previous *in vitro* study showing that PolB prefers gap-filling type substrates to primer-extension type substrates, while the substrate preference of PolD is the opposite, supports this prediction^30^. We have also confirmed that the PolB of *P. furiosus* has strand displacement activity *in vitro* (Kimizu *et al.*, unpublished result). Taken together with these results, strand displacement DNA synthesis by PolB, cleavage of the resultant flapped DNA by Fen1 endonuclease, and nick-sealing by DNA ligase will occur after incision by EndoQ, as in the case of the BER pathway. PCNA will have an important role to provide a scaffold for EndoQ, PolB, Fen1 and Lig to work on DNA efficiently for their sequential tasks (Fig. 5). Further analyses will elucidate this prediction of the damaged base repair process in the *Thermococcales*. It is also of evolutionary interest that the *endoQ* gene is not found in the hyperthermophilic archaeal subdomain of Crenarchaeota, which includes organisms such as *Sulfolobus solfataricus*, *Sulfolobus islandicus*, *Aeropyrum pernix* and *Pyrodictium occultum*. However, the gene is conserved in the methanogenic archaea, suggesting that this gene was likely acquired or invented in the archaeal subdomain Euryarchaeota, which includes the methanogens (hyperthermophilic, thermophilic and mesophilic), the halophiles, and the *Thermococcales*. The presence of the gene in some bacteria is not surprising, as the methanogens tend to grow in association with bacteria in many environments including the soil and mammalian guts, and this important gene can be acquired through horizontal gene transfer. We are currently investigating the function of the EndoQ homologs in both the mesophilic and hyperthermophilic methanogens to help shed more light on the evolution and distribution of this very fascinating DNA repair enzyme.

In conclusion, we presented here the physical and functional interactions between EndoQ and PCNA. EndoQ is probably acquired for the efficient repair of damaged bases in hyperthermophilic archaea and evolved in the archaeal and bacterial domains to form a repairsome with PCNA.

## Methods

### Clones and proteins

The genes encoding the TkoEndoQ and PfuEndoQ with their C-terminal truncation (deletion of residues 409–421 and 411–424, respectively), designated ∆PIP, and TkoPCNA1 with mutations at E143A/D147A were generated by site-specific mutagenesis. We designed the TkoPCNA1 mutant that does not form a stable ring structure in solution based on the previous works^17^,^31^. The PCR reaction mixtures (25 μl) contained 25 ng pET-TK0887 plasmid for TkoEndoQ, pET-PF1551 for PfuEndoQ^6^, or pET-TK0535 for TkoPCNA1^18^, 1 × PCR buffer for KOD-Plus-Neo DNA polymerase (TOYOBO, Osaka, Japan), 1.5 mM Mg_2_SO_4_, 0.2 mM of each dNTP, 0.3 μM primers (TkoEndoQ^∆PIP^, 5′-ACGTTGAGGAAAAGCCCAAGTGAAGGAGC ATAACCGAATTCCT and 5′-AGGAATTCGGTTATGCTCCTTCACTTGGGCTTTTCCTCAACGT; PfuEndoQ^∆PIP^, 5′-CGAGTTGCCGAAACCTAAGTGAAGGA-CCCTGCTTCAATATATT and 5′-AATATATTGAAGCAGGGTCCTTCACTTAGGTTTCGGCAACTCG, stop codons are underlined) (TkoPCNA1E143A/D147A 5′-GTGAGGTTCTCAAGGCCGGCATAAAGGCCGCTTCCCTCGTCAG and 5′-CTGACGAGGGAAGCGGCCTTTATGCCGGCCTTGAGAACCTCAC), and 0.5 unit KOD-Plus-Neo DNA polymerase (TOYOBO, Osaka, Japan). The mixtures were heated at 95 °C for 30 s and then subjected to thermal cycling (14 cycles of 95 °C for 10 s, 55 °C for 30 s, and 68 °C for 5 min). The PCR products were treated with DpnI (NEB) at 37 °C for 1 h, and transformed into *E. coli* JM109 cells. Each full insert was sequenced to verify the targeted mutation. The expression and purification of TkoEndoQ^WT^, TkoEndoQ^ΔPIP^, and TkoPCNA1 were performed as described previously^6^,^18^. TkoPCNA1^E143A/D147A^ was also purified basically as same as the TkoPCNA1^WT^, however, we used a 5 ml HiTrap Heparin HP column (GE Healthcare) and a 1 ml MonoQ 5/50 column (GE Healthcare). Then protein concentrations were calculated by measuring the absorbance at 280 nm. The theoretical molar extinction coefficients of TkoEndoQ^WT^, TkoEndoQ^ΔPIP^, TkoPCNA1^WT^, TkoPCNA1^E143A/D147A^, PfuEndoQ^WT^, PfuEndoQ^ΔPIP^, and PfuPCNA^WT^ were calculated as 48610, 48610, 5960, 5960, 47120, 45630, and 7450 M^−1^ cm^−1^, respectively.

### Surface Plasmon Resonance (SPR) analysis

A Biacore J (GE healthcare) system was used to study the physical interaction between TkoEndoQ and TkoPCNA1. Purified recombinant TkoPCNA1 were bound to CM5 sensor chip according to the manufacturer’s recommendation. To measure the kinetic parameters, various concentrations of TkoEndoQ (12, 25, 50, 75, 100, 400 and 800 nM) were applied to the immobilized TkoPCNA1. All experiments were conducted at 25 °C in a buffer containing 10 mM HEPES, pH7.4, 0.4 M NaCl and 0.005% Tween20. Regenerations at the end of each cycle were achieved by injections of 2 M NaCl. The equilibrium dissociation constants (*K*_D_) were determined from the association and dissociation curves of the sensorgrams, using the BIAevaluation program (GE healthcare).

### DNA substrates and cleavage assay

The deoxyinosine (dI)-containing oligonucleotide (45-I25, 5′-dCGAACTGCCTGGAATCCTGACGACITGTAGCGAACGATCACCTCA), labeled by Cy5 at the 5′ terminus and its complementary oligonucleotide (45R, 5′-dTGAGGTGATCGTTCGCTACATGTCGTCAGGATTCC- AGGCAGTTCG) were obtained from Sigma Aldrich (Tokyo, Japan) Double-stranded DNA was prepared by annealing 45-I25 and 45R in TAM buffer (40 mM Tris-acetate, pH 7.8 and 0.5 mM Mg(CH_3_COO)_2_). The cleavage reactions were performed at 75 °C for 10 min in a 20 μl reaction mixture, containing 50 mM Tris-HCl, pH8.0, 1 mM DTT, 1 mM MgCl_2_, 0.01% Tween20, 0.4 M NaCl, 5 nM DNA substrate, 10 nM TkoEndoQ and various concentrations of TkoPCNA1 (0, 0.18, 0.6, and 1.8 μM, as a monomer). Reactions were terminated with 40 μl of stop solution (98% deionized formamide, 10 mM EDTA and 0.1% OrangeG). After an incubation at 95 °C for 5 min, the samples were immediately placed on ice. The samples were separated by 8 M urea-12% PAGE in TBE buffer (89 mM Tris-borate and 2.5 mM EDTA). The gel image was visualized and the resulting bands were quantified with a Typhoon image analyzer (GE healthcare).

## Additional Information

**How to cite this article**: Shiraishi, M. *et al.* PCNA is involved in the EndoQ-mediated DNA repair process in *Thermococcales*. *Sci. Rep.* **6**, 25532; doi: 10.1038/srep25532 (2016).

## Supplementary Material

Supplementary Information

## References

[b1] Lindahl T. Instability and decay of the primary structure of DNA. Nature 362, 709–715 (1993). 8469282 10.1038/362709a0

[b2] Barnes D. E. & Lindahl T. Repair and genetic consequences of endogenous DNA base damage in mammalian cells. Annu. Rev. Genet. 38, 445–476 (2004). 15568983 10.1146/annurev.genet.38.072902.092448

[b3] Berti P. J. & McCann J. A. Toward a detailed understanding of base excision repair enzymes: transition state and mechanistic analyses of N-glycoside hydrolysis and N-glycoside transfer. Chem. Rev. 106, 506–555 (2006). 16464017 10.1021/cr040461t

[b4] Aravind L. & Koonin E. V. The alpha/beta fold uracil DNA glycosylases: a common origin with diverse fates. Genome Biol. 2000/1, research 0007.1-0007.8 (2000). 10.1186/gb-2000-1-4-research0007 PMC15025 11178247

[b5] Pearl L. H. Structure and function in the uracil-DNA glycosylase superfamily. Mutat. Res. 460, 165–181 (2000). 10946227 10.1016/s0921-8777(00)00025-2

[b6] Shiraishi M. *et al.* A novel endonuclease that may be responsible for damaged DNA base repair in *Pyrococcus furiosus*. Nucleic Acids Res. 43, 2853–2863 (2015). 25694513 10.1093/nar/gkv121 PMC4357722

[b7] Kiyonari S., Egashira Y., Ishino S. & Ishino Y. Biochemical characterization of endonuclease V from the hyperthermophilic archaeon. Pyrococcus furiosus. J. Biochem. 153, 325–333 (2014). 24535600 10.1093/jb/mvu010

[b8] Ishino S., Makita N., Shiraishi M., Yamagami T. & Ishino Y. EndoQ and EndoV work individually for damaged DNA base repair in *Pyrococcus furiosus*. Biochimie 118, 264–269 (2015). 26116888 10.1016/j.biochi.2015.06.015

[b9] Moldovan G. L., Pfander B. & Jentsch S. PCNA, the maestro of the replication fork. Cell 129, 665–679 (2007). 17512402 10.1016/j.cell.2007.05.003

[b10] Dalrymple B. P., Kongsuwan K., Wijffels G., Dixon N. E. & Jennings P. A universal protein-protein interaction motif in the eubacterial DNA replication and repair systems. Proc. Natl. Acad. Sci. USA 98, 11627–11632 (2001). 11573000 10.1073/pnas.191384398 PMC58780

[b11] Warbrick E. M. The puzzle of PCNA’s many partners. BioEssays 22, 997–1006 (2000). 11056476 10.1002/1521-1878(200011)22:11<997::AID-BIES6>3.0.CO;2-#

[b12] Vivona J. B. & Kelman Z. The diverse spectrum of sliding clamp interacting proteins. FEBS Lett. 546, 167–167 (2003). 12832034 10.1016/s0014-5793(03)00622-7

[b13] Kiyonari S., Uchimura M., Shirai T. & Ishino Y. Physical and functional interactions between uracil-DNA glycosylase and proliferating cell nuclear antigen from the euryarchaeon *Pyrococcus furiosus*. J. Biol. Chem. 283, 24185–24193 (2008). 18562313 10.1074/jbc.M802837200 PMC3259797

[b14] Kiyonari S. *et al.* Biochemical properties and BER complex formation of AP endonuclease from *Pyrococcus furiosus*. Nucleic Acids Res. 37, 6439–6453 (2009). 19734344 10.1093/nar/gkp720 PMC2770678

[b15] Yang H. *et al.* Direct interaction between uracil-DNA glycosylase and a proliferating cell nuclear antigen homolog in the crenarchaeon *Pyrobaculum aerophilum*. J. Biol. Chem. 277, 22271–22278 (2002). 11927597 10.1074/jbc.M201820200

[b16] Dionne I. & Bell S. D. Characterization of an archaeal family 4 uracil DNA glycosylase and its interaction with PCNA and chromatin proteins. Biochem. J. 387, 859–863 (2005). 15588253 10.1042/BJ20041661 PMC1135018

[b17] Matsumiya S., Ishino S., Ishino Y. & Morikawa K. Intermolecular ion pairs maintain toroidal structure of *Pyrococcus furiosus* PCNA. Prot. Sci. 12, 823–831 (2003). 10.1110/ps.0234503 PMC2323854 12649440

[b18] Kuba Y. *et al.* Comparative analyses of the two PCNAs from the hyperthermophilic archaeon, Thermococcus kodakarensis. Genes Cells 17, 923–937 (2012). 23078585 10.1111/gtc.12007

[b19] Pan M. *et al.* *Thermococcus kodakarensis* has two functional PCNA homologs but only one is required for viability. Extremophiles 17, 453–461 (2013). 23525944 10.1007/s00792-013-0526-8 PMC3743106

[b20] Kiyonari S. *et al.* Studies on base excision repair (BER) complex in *Pyrococcus furiosus*. Biochem. Soc. Trans. 37, 79–82 (2009). 19143606 10.1042/BST0370079

[b21] Parlanti E., Locatelli G., Maga G. & Dogliotti E. Human base excision repair complex is physically associated to DNA replication and cell cycle regulatory proteins. Nucleic Acids Res. 35, 1569–1577 (2007). 17289756 10.1093/nar/gkl1159 PMC1865045

[b22] Lasken R. S., Schugter D. M. & Rashtchian A. Archaebacterial DNA polymerases tightly bind uracil-containing DNA. J. Biol. Chem. 271, 17692–17696 (1996). 8663453 10.1074/jbc.271.30.17692

[b23] Greagg M. A. *et al.* A read-ahead function in archaeal DNA polymerases detects promutagenic template-strand uracil. Proc. Natl. Acad. Sci. USA 96, 9045–9050 (1999). 10430892 10.1073/pnas.96.16.9045 PMC17729

[b24] Fogg M. J., Pearl L. H. & Connolly B. A. Structural basis for uracil recognition by archaeal family B DNA polymerases. Nat. Struct. Biol. 9, 922–927 (2002). 12415291 10.1038/nsb867

[b25] Hogrefe H. H., Hansen C. J., Scott B. R. & Nielson K. B. Archaeal dUTPase enhances PCR amplifications with archaeal DNA polymerases by preventing dUTP incorporation. Proc. Natl. Acad. Sci. USA 99, 596–601 (2002). 11782527 10.1073/pnas.012372799 PMC117351

[b26] Connolly B. A., Fogg M. J., Shuttleworth G. & Wilson B. T. Uracil recognition by archaeal family B DNA polymerases. Biochem. Soc. Trans. 31, 699–702 (2003). 12773186 10.1042/bst0310699

[b27] Kiyonari S., Takayama K., Nishida H. & Ishino Y. Identification of a novel binding motif in *Pyrococcus furiosus* DNA ligase for the functional interaction with proliferating cell nuclear antigen. J. Biol. Chem. 281, 28023–28032 (2006). 16829513 10.1074/jbc.M603403200

[b28] Ishino S. & Ishino Y. Comprehensive search for DNA polymerase in the hyperthermophilic archaeon, *Pyrococcus furiosus*. Nucleosides Nucleotides Nucleic Acids, 25, 681–691 (2006). 16838855 10.1080/15257770600686485

[b29] Cubonová L. *et al.* Archaeal DNA polymerase D but not DNA polymerase B is required for genome replication in *Thermococcus kodakarensis*. J. Bacteriol. 195, 2322–2328 (2013). 23504010 10.1128/JB.02037-12 PMC3650531

[b30] Ishino Y. & Ishino S. DNA polymerases from euryarchaeota. Methods Enzymol. 334, 249–260 (2001). 11398467 10.1016/s0076-6879(01)34473-7

[b31] Kawamura A., Ishino Y. & Ishino S. Biophysical analysis of PCNA from *Pyrococcus furiosus*. J. Jap. Soc. Extremophiles. 11, 12–18 (2012).

