# Supplementary material for: PCNA is involved in the EndoQ-mediated DNA repair process in Thermococcales
Source: Sci Rep. 2016 May 6;6:25532. doi: 10.1038/srep25532 (PMC4858679; doi:10.1038/srep25532)
Supplement: Supplementary Information [file srep25532-s1.pdf]

Supplementary Information

PCNA is involved in the EndoQ-mediated DNA repair process in *Thermococcales*

Miyako Shiraishi[1, 4, 5], Sonoko Ishino[1], Kotaro Yoshida[1], Takeshi Yamagami[1], Isaac Cann[2, 3, 4, 5] Yoshizumi Ishino[1, 4, 5*]

[1]Graduate School of Bioresource and Bioenvironmental Sciences, Kyushu University, Fukuoka, Japan, [2]Department of Animal Science, and [3]Department of Microbiology, [4]Institute for Universal Biology and [5]Carl R. Woese Institute for Genomic Biology, University of Illinois at Urbana-Champaign, Urbana, Illinois, USA
* E-mail: ishino@agr.kyushu-u.ac.jp

β-clamp consensus motif in Bacteria			Q	L	D<br>S	L	F	
Species	Accession							
Thermodesulfovibrio thiophilus	WP_028844875	413	Q	T	T	L	F	417
Candidatus Aminicenans sakinawicola	WP_020259138	426	Q	L	K	L	F	430
Dictyoglomus thermophilum	WP_012548172	401	Q	I	S	L	F	405
Parcubacteria bacterium GW2011_GWA1_36_12	KKP93828	412	Q	K	S	L	F	416
Geobacter sulfurreducens	WP_041914077	422	Q	G	G	L	F	426
Syntrophorhabdus aromaticivorans	WP_038003961	419	Q	A	T	L	F	423
Microgenomates (Daviesbacteria) bacterium GW2011_GWA2_38_24	KKQ65637	424	Q	V	G	L	F	428
Microgenomates (Woesebacteria) bacterium GW2011_GWA1_39_12	KKQ98233	460	Q	Q	A	L	F	464
Thermodesulfatator atlanticus	WP_022853876	421	Q	K	S	L	F	425
Thermodesulfatator indicus	WP_013907803	420	Q	K	S	L	F	424
Desulfobacter curvatus	WP_020586011	1084	Q	L	E	L	F	1088
Desulfobacterium autotrophicum	WP_015906045	1152	Q	L	D	L	F	1156
Proteiniphilum acetatigenes	WP_019538689	1053	Q	L	R	L	F	1057
Cloacimonetes bacterium SCGC AAA252-K16	WP_029157127	1069	Q	Q	Q	L	F	1073
Cloacimonetes bacterium JGI 0000014-D18	WP_029156197	1068	Q	M	R	L	F	1072
Desulfomonile tiedjei	WP_014810252	977	Q	C	D	L	F	981
Caldilinea aerophila	WP_014432234	1130	Q	L	G	L	F	1134
Rhodococcus rhodnii	WP_037252411	1017	Q	L	R	L	L	1021
Saccharopolyspora spinosa	WP_029535017	1033	Q	L	T	L	L	1037
Opitutaceae bacterium TAV5	WP_009508792	1067	Q	L	D	L	F	1071
Diplosphaera colitermitum	WP_043584854	1065	Q	L	D	L	F	1069
Sorangium cellulosum	WP_012236160	1085	Q	M	K	L	F	1089
Chondromyces apiculatus	WP_044237261	1105	Q	L	K	L	F	1109
Haliangium ochraceum	WP_012825435	1157	Q	L	G	L	F	1161
Rhodothermus marinus	WP_014066013	1028	Q	L	E	L	F	1032
Elioraea tepidiphila	WP_019013748	1011	Q	P	S	L	F	1015
Pleomorphomonas koreensis	WP_026782320	1030	Q	L	S	L	F	1034
Inquilinus limosus	WP_034836501	1052	Q	L	S	L	F	1056
Candidatus Achromatium palustre	WP_048399368	1133	Q	L	D	L	F	1137
Fusobacterium sp. CAG:439	CDE59466	956	Q	L	S	L	F	960
Acinetobacter sp. CAG:196	CCZ49697	941	Q	L	S	L	F	945
Brachyspira sp. CAG:484	CCY23933	973	Q	L	S	L	F	977
Zymomonas mobilis	WP_011241259	1043	Q	Y	S	L	F	1047
Geobacter metallireducens	WP_004512662	1071	Q	L	E	L	F	1075
Desulfobulbus japonicus	WP_028582461	1092	Q	L	S	L	F	1096
Clostridiales bacterium 1_7_47FAA	WP_008716654	1113	Q	L	S	L	F	1117
Spirochaeta thermophila	WP_014625139	1004	Q	Y	T	L	F	1008
Desulfovibrio sp. X2	WP_020880034	1078	Q	I	Q	L	M	1082
Desulfovibrio vulgaris	WP_012612878	1240	Q	L	S	L	M	1244
Desulfovibrio desulfuricans	WP_014321782	1027	Q	L	G	L	L	1031
Desulfovibrio salexigens	WP_015852834	1028	K	I	S	L	L	1032
Desulfovibrio gigas	WP_021759146	1067	T	L	S	L	L	1071
Bilophila wadsworthia	WP_005028539	1071	Q	M	K	L	F	1075
Treponema azotonutricium	WP_043922803	1070	Q	L	R	L	F	1074

Supplementary Figure S1. Putative β-clamp binding motifs in bacterial EndoQ homologs. The sequences were aligned by ClustalW2 (http://www.ebi.ac.uk/Tools/msa/clustalw2/). Positions of the motifs are indicated by the amino acid number on the left (start) and right (end) of the sequences. The consensus sequence of the β-clamp binding motif in Bacteria (QL[D/S]LF) is shown on the top of the panel. Conserved amino acids with the consensus motif are colored by cyan background.

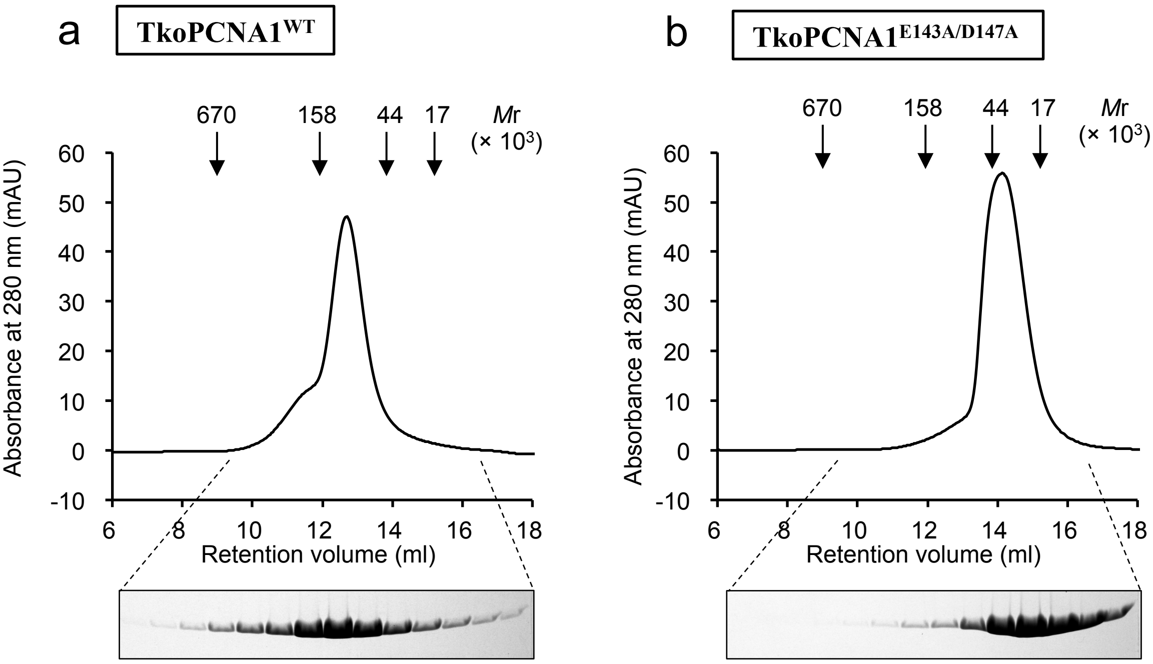

Supplementary Figure S2. Analysis of the oligomeric states of TkoPCNA1^{WT} and TkoPCNA1$^{E143A/D147A}$ in solution by gel filtration. The WT (a) and E143A/D147A mutant (b) proteins (0.5 mg/ml in 500 μl) were subjected to gel filtration analyses with a Superdex200 10/300 GL column (GE healthcare) in 50 mM Tris-HCl, pH8.0, 0.5 mM DTT, 0.1 mM EDTA, 10 % glycerol and 0.15 M NaCl using an AKTA FPLC system (GE healthcare). Elution of the protein was monitored by the absorbance at 280 nm. The peak positions of the marker proteins are indicated on the top. The relative molecular masses of the PCNAs were estimated by a standard curve obtained from a Gel Filtration Standard (Bio-Rad; γ-globulin (158,000), ovalbumin (44,000) and myoglobin (17,000)) in the same buffer. The estimated molecular weights of TkoPCNA1^{WT} and TkoPCNA1$^{E143A/D147A}$ were 99.1 k and 37.3 k, respectively. The peak fractions were analyzed by SDS-12% PAGE, followed by Coomassie brilliant blue (CBB) staining (bottom of each elution profile).

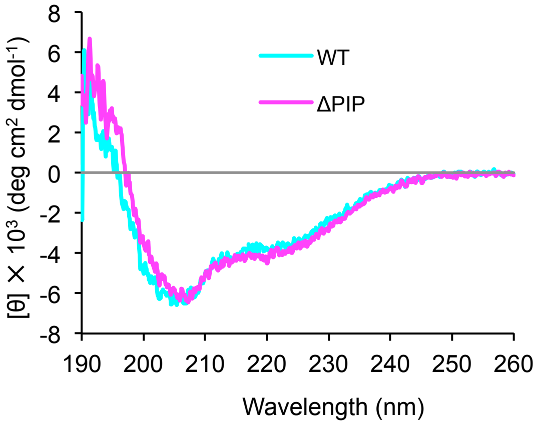

Supplementary Figure S3. Structural comparison of TkoEndoQ^{WT} and TkoEndoQ$^{\Delta PIP}$ by CD spectrometry. The proteins dissolved at 0.1 mg/ml in 10 mM sodium phosphate, pH7.0, were subjected to CD scan by J815-150S Spectropolarimeter (JASCO) at 25°C from 190 nm to 260 nm. The spectra were collected at a scan speed of 20 nm/min, a time constant of 1 s, and a bandwidth of 1 nm. Five scans were accumulated and representative scans were shown in cyan (TkoEndoQ^{WT}) and in magenta (TkoEndoQ$^{\Delta PIP}$).

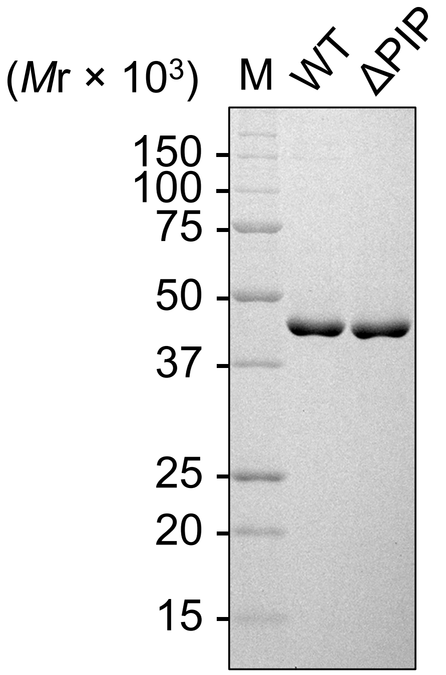

Supplementary Figure S4. Purification of the recombinant PfuEndoQ proteins. The protein marker (Bio-Rad; lane M) and 2 μg of each purified protein (PfuEndoQ^{WT} and PfuEndoQ$^{\Delta PIP}$) were subjected to SDS-12% PAGE followed by CBB staining. The sizes of the marker proteins are shown on the left of the panel.

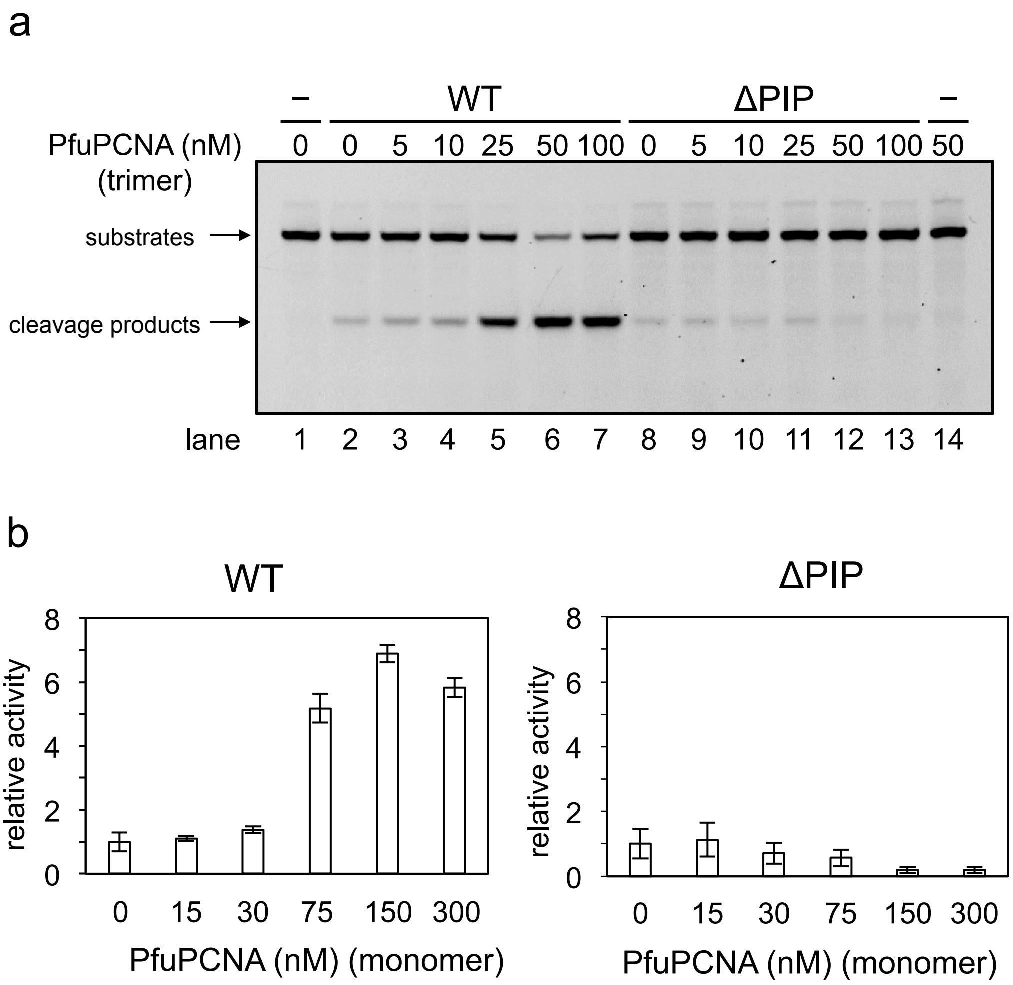

Supplementary Figure 5. Cleavage activity of PfuEndoQ^{WT} and PfuEndoQ$^{\Delta PIP}$ in the presence and absence of PfuPCNA. (a) 5′-Cy5-labeled 49-I25 dsDNA substrates (10 nM) were incubated at 75˚C for 5 min in a 20 µl reaction solution containing 50 mM Tris–HCl, pH 8.0, 180 mM NaCl, 1 mM DTT, 1 mM $MgCl_2$, 0.01% Tween 20. −, no EndoQ (lane 1 and 14); WT, 2 nM PfuEndoQ^{WT} (lane 2–7); 2 nM PfuEndoQ$^{\Delta PIP}$ (lane 8–13); the concentration of PfuPCNA was varied as indicated. Cleavage products were separated by 8 M urea–12% PAGE and visualized by an image analyzer, Typhoon Trio+ (GE Healthcare).

(b) Resulting band intensities from Supplementary Figure 5a experiments were quantified with ImageQuant TL (GE healthcare). The relative cleavage activities with the standard errors from three independent experiments are shown.

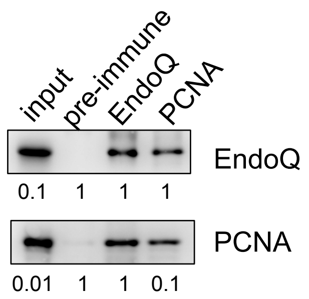

Supplementary Figure S6. Coexistence of EndoQ and PCNA in *P. furious cells*. *P. furiosus* cells were cultured in 100 ml of medium at 98°C, and were harvested at the exponential growth phase (A_{600} = 0.45). The cells (3 × 10^{10}) were suspended and disrupted in 1.5 ml lysis buffer (50 mM Tris–HCl, pH 8.0, 50 mM NaCl, 0.5 mM DTT, 0.1 mM EDTA, 10% glycerol, and 0.1% Tween-20) by sonication. A portion (50 μl) of rProtein A Sepharose FF (GE healthcare) was washed twice with PBS–T (10 mM sodium phosphate, pH 7.5, 150 mM NaCl, and 0.1% Tween 20), and mixed with 25 μl of anti-TkoEndoQ, or anti-PfuPCNA antiserum, and incubated at room temperature for 1 h. Each mixture was washed twice with PBS–T, and then twice with 0.2 M triethanolamine, pH 8.0. The antibody was cross-linked to the rProtein A Sepharose FF with dimethyl pimelimidate 2 HCl (DMP, Thermo Scientific Pierce), according to the manufacturer's protocol. Pre-immune serum was used for negative control experiments. After equilibration of the antibody-conjugated rProtein A Sepharose FF with lysis buffer, an aliquot of the cell extract (400 μl, 0.8 × 10^{10} cells) was added, and the mixture was incubated at room temperature for 1 h. The precipitates were washed thrice with lysis buffer, and the immunoprecipitated proteins were eluted from the beads with 40 μl of gel loading solution (50 mM Tris–HCl, pH 6.8, 10 % glycerol, 100 mM DTT, 0.2 mg/ml bromophenol blue, 2% SDS) at 98°C for 3 min. The eluates were subjected to SDS-12% PAGE, followed by western blot analysis. The relative amounts of loading are indicated at the bottom for each band. The loading amount was adjusted to avoid saturation of the protein band intensities. The antisera used for precipitation and detection are indicated at the top and the side, respectively.
